# Individual placement and support (IPS) for patients with offending histories: the IPSOH feasibility cluster randomised trial protocol

**DOI:** 10.1136/bmjopen-2016-012710

**Published:** 2016-07-22

**Authors:** N Khalifa, E Talbot, J Schneider, D M Walker, P Bates, Y Bird, D Davies, C Brookes, J Hall, B Völlm

**Affiliations:** 1 Nottinghamshire Healthcare NHS Foundation Trust, Nottingham, UK; 2 University of Nottingham, Nottingham, UK; 3 Institute of Mental Health, Nottingham, UK; 4 Faculty of Health Sciences, University of Southampton, Southampton, UK; 5 Patient and Public Involvement Lead, Nottingham, UK; 6 Leicestershire and Rutland Probation Trust, Leicestershire, UK; 7 Leicester Clinical Trials Unit, University of Leicester, Leicester, UK

**Keywords:** Individual placement and support, Supported Employment, Forensic Mental Health, Offenders, MENTAL HEALTH

## Abstract

**Introduction:**

People with involvement in forensic psychiatric services face many obstacles to employment, arising from their offending, as well as their mental health problems. This study aims to assess the feasibility of conducting a randomised controlled trial (RCT) to evaluate the effectiveness of individual placement and support (IPS), in improving employment rates and associated psychosocial outcomes in forensic psychiatric populations. IPS has been found consistently to achieve employment rates above 50% in psychiatric patients without a history of involvement in criminal justice services.

**Methods/design:**

This is a single-centre feasibility cluster RCT. Clusters will be defined according to clinical services in the community forensic services of Nottinghamshire Healthcare NHS Foundation Trust (NHCT). IPS will be implemented into 2 of the randomly assigned intervention clusters in the community forensic services of NHCT. A feasibility cluster RCT will estimate the parameters required to design a full RCT. The primary outcome is the proportion of people in open employment at 12-month follow-up. Secondary outcome measures will include employment, educational activities, psychosocial and economic outcomes, as well as reoffending rates. Outcome measures will be recorded at baseline, 6 months and 12 months. In accordance with the UK Medical Research Council guidelines on the evaluation of complex interventions, a process evaluation will be carried out; qualitative interviews with patients and staff will explore general views of IPS as well as barriers and facilitators to implementation. Fidelity reviews will assess the extent to which the services follow the principles of IPS prior, during and at the end of the trial.

**Ethics and dissemination:**

Ethical approval was obtained from the East Midlands Research Ethics Committee-Nottingham 1 (REC reference number 15/EM/0253). Final and interim reports will be prepared for project funders, the study sponsor and clinical research network. Findings will be disseminated through peer-reviewed journals, conferences and event presentations.

**Trial registration number:**

NCT02442193; Pre-results.

**Strengths and limitations of this study**

- This study will be the first in the UK to consider how best individual placement and support (IPS) can be adapted for patients with offending histories.
- Our study will provide commissioners with a full understanding of how IPS could be implemented in forensic mental health settings elsewhere and provide information about the key parameters needed to design a full RCT.
- This study is a small-scale, single-centre study. The study is a feasibility trial and as such does not allow us to draw conclusions about the efficacy of IPS.

## Introduction

Unemployment has a number of undesirable consequences and has been linked to low personal well-being,^1^ poor physical and/or mental health^2^ and can serve to exacerbate these pre-existing health conditions.^3^ Besides income, work is also widely recognised as being beneficial to an individual's mental health^2^ ^4^ and can enhance a person's self-esteem and inspire optimism.^4^

People with severe mental disorders have far higher rates of unemployment when compared with the general population^5^ and are more likely to be unemployed compared with the general disabled population.^6^ Recent Department for Work and Pensions’ (DWP) figures show that 6.5 million working-age people in the UK are classified as disabled under the Equality Act 2010 because they have a physical or mental condition that affects their ability to carry out daily activities.^7^ However, many of these people can work and the employment aspirations of many remain unfulfilled. Use of supported employment, to help with this issue, has been consistently advocated in public policy.^7^

Furthermore, employment rates for offenders are also worryingly low, with the Ministry of Justice and DWP^8^ reporting that 29% of prisoners found paid employment at some point in the 2 years following their release and that only 15% were in paid employment 2 years after their release. Government initiatives have also emphasised the importance of using work to reduce reoffending rates among offenders.^9^ Offending history is further compounded by the fact that the prevalence of mental disorders among offenders is far greater than in the general population.^10^

Offenders with mental disorders arguable fare worse in regards to employment partly due to the stigma attributed to their offending history and negative stereotypes of people with mental health problems on the part of employers and members of the public.^11^ Complex personal and social problems, including homelessness, substance misuse, lack of relevant skills or qualifications and limited employment experience, make it even more difficult for them to secure employment.^12^

There is strong evidence in favour of individual placement and support (IPS), a well-established form of supported employment, as the most effective approach to help people with severe mental disorders get back into employment.^5^ ^13–17^ IPS operates according to the following principles: (1) it aims to get people into competitive employment; (2) it is open to all those who want to work; (3) it tries to find jobs consistent with people's preferences; (4) it works quickly; (5) it brings employment specialists into clinical teams; (6) employment specialists develop relationships with employers based on a person's work preferences; (7) it provides time unlimited, individualised support for the person and their employer; and (8) benefits counselling is included.

The Cochrane review by Kinoshita *et al*,^5^ including 14 randomised controlled trials (RCTs) (2265 participants in total), showed that IPS significantly increased levels of employment and increased length of competitive employment for individuals with severe mental illness, when compared with other vocational approaches. Although the literature supports the effectiveness of IPS in generic mental healthcare settings, the evidence base for its effectiveness in forensic mental health settings is lacking.^18^ A paper commissioned by the Sainsbury Centre for Mental Health,^19^ which reviewed the literature on employment support for offenders with mental health problems, concluded that many principles of IPS, if suitably adapted, have the potential to help offenders with mental disorders gain employment. However, it is not known how best to adapt IPS to forensic mental health settings, and to the best of our knowledge, no empirical studies of IPS in UK community forensic mental health settings have been completed to date.

IPS can be regarded as a complex intervention as it involves a number of interacting components. Developing an evidence base for IPS in a forensic mental health setting adds to this complexity, since the management of mentally disordered offenders combines various treatment modalities to address mental health issues, offending behaviour and risk management.^20^ Nevertheless, little attention, particularly in the health sector, is paid to supporting mentally disordered offenders into mainstream employment. It is the very complexities in the practice of forensic mental health, which make the implementation and evaluation of IPS a challenge; the present study is, therefore, needed to underpin robust future RCTs, so that this intervention, proven to be effective in adults with mental health problems, can be appraised for its potential to enable patients with offending histories to live more rewarding lives, reduce reoffending and minimise their dependency on formal services. It is hypothesised that the benefits of IPS found in adult mental health settings will be replicated to some degree in the forensic population. The proposed study will, therefore, bring benefits to patients, to the local service, to national forensic and mental health services and will inform commissioning and policymakers.

## Aims and objectives

The overall aim of the study is to assess the feasibility of conducting a fully powered RCT to evaluate the effectiveness of IPS in improving employment rates and associated psychosocial outcomes in community forensic psychiatric populations.

The specific objectives of the study are as follows:

1. To estimate the parameters required to design a full RCT, including means and SDs, of the key outcome measures in order to benchmark potential effect sizes and enable sample size calculations.
2. To assess the feasibility of randomisation, recruitment and retention rates to the IPS and control arms.
3. To explore the suitability of the key outcome measures with respect to reliability, acceptability and distribution of the scores.

A process evaluation will also be carried out; qualitative interviews with patients and staff will explore general views of IPS as well as barriers and facilitators to implementation, while fidelity reviews will assess the extent to which the services follow the principles of IPS.

## Methods/design

The design of this study has drawn from the principles set out in the Medical Research Council Guidance^21^ on developing and evaluating complex interventions by using three major strands with clearly defined time scales as follows.

### Implementation of IPS in community forensic services (6 months)

A fundamental prerequisite to IPS research is appropriate implementation. This study will, therefore, allocate 6 months to embedding the IPS model within the intervention clusters. IPS will be embedded by bringing an employment specialist into the clinical teams, raising awareness about IPS within the organisation and forming links with IPS services within the local National Health Service (NHS) Trust, in addition to developing important links with employers and the DWP. This process of implementation is guided by prior work in adult mental health and led by a dedicated development team.^22^

### Feasibility study (24 months)

#### Participants and study design

This study will recruit adult patients (male and female) who are over the age of 18 and who are currently on the caseload of the community forensic services. Patients who are unable to provide informed consent, who are not eligible to work in the UK, do not have an offending history, are currently in open employment or who do not wish to work will be excluded from this study.

This study employs a cluster RCT design. Clusters are defined according to clinical services in the community forensic services, which include four major divisions as follows: Cluster 1: City Community Forensic Service. Cluster 2: County Community Forensic Service. Cluster 3: City Personality Disorder Service. Cluster 4: County Personality Disorder Service.

#### Randomisation: sequence generation

**Figure 1:**
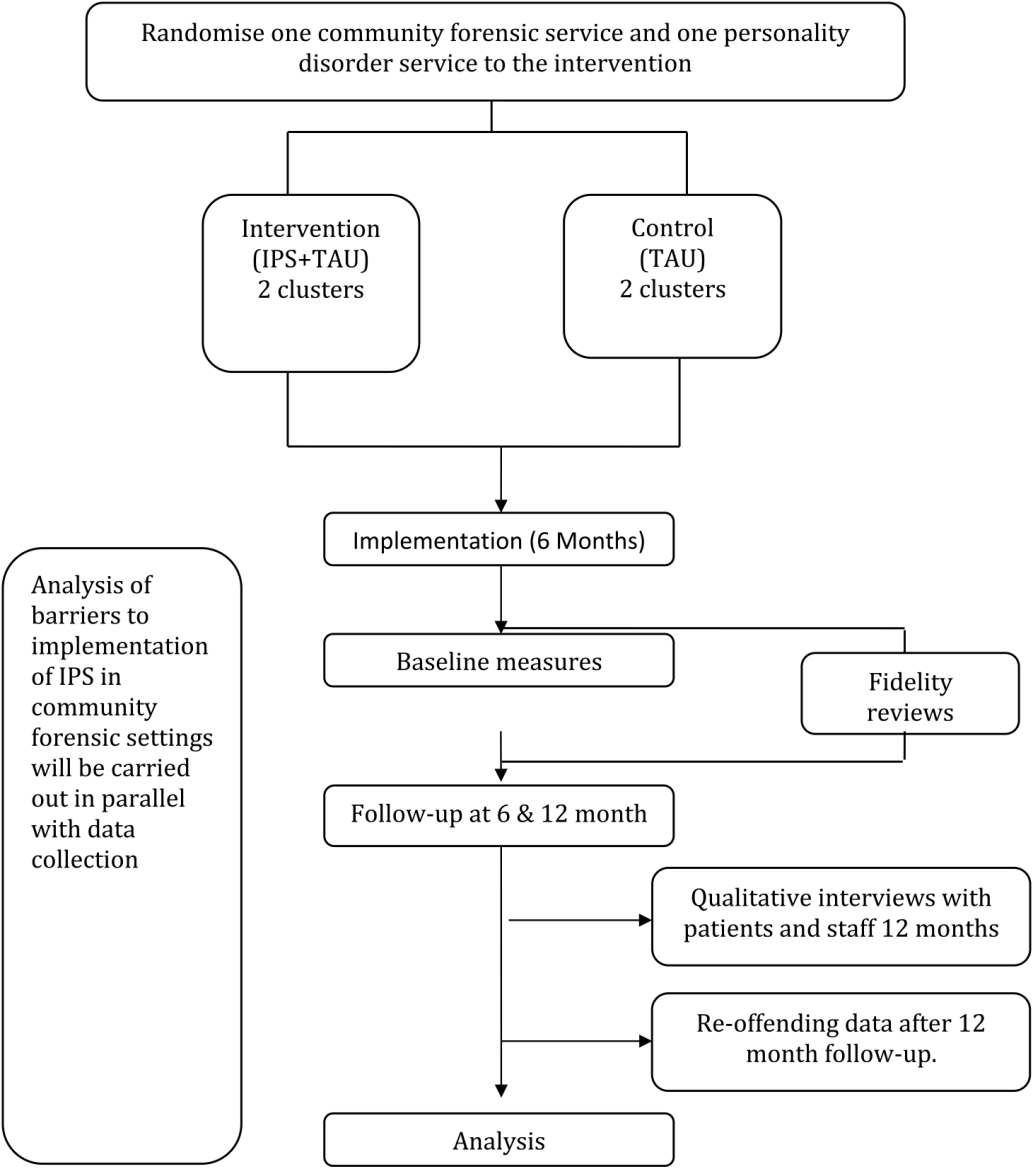
Trial flow chart. IPS, individual placement and support; TAU, treatment as usual.

Randomisation of clusters was carried out by the trial statistician from the University of Leicester Clinical Trials Unit, allocating two clusters (City Community Forensic Service and the County Personality Disorder Service) to the intervention group (IPS+treatment as usual [TAU]). The remaining two clusters (County Community Forensic Service and City Personality Disorder Service) were allocated to the control group (TAU) (figure 1 for trial flow chart).

#### Interventions

Group 1: Individuals assigned to the intervention group will receive IPS+TAU. Individuals will receive ongoing support from the employment specialist in accordance with the IPS principles. The employment specialist will work closely with patients assigned to IPS and begin job searches rapidly, based on their preferences. Colocation of the employment specialist within clinical teams will allow information about risks and past offending histories to be shared with employers (with patient's consent), health and criminal justice systems (for those concurrently under probation) and subsequently taken into consideration when matching job opportunities to individual preferences. The employment specialist will bridge the gap between the research team and the community forensic teams by virtue of being a member of both teams.

Group 2: Individuals assigned to the control group will not receive any IPS support but will continue to receive TAU from the community forensic services. TAU for patients who are part of the city or county forensic teams entails case management, where each individual is allocated a consultant and care coordinator. TAU for patients who are part of the City or County Personality Disorder teams will primarily be made up of therapy services providing specialised psychological interventions, including group therapy and attendance, at a therapeutic community. Neither groups are offered specialist employment support as part of TAU.

#### Sample size

The sample size calculation was based on an assertion by Eldridge and Kerry^23^ that, for samples of 75–150 individuals, four clusters will be required to achieve reasonably precise 95% CI for intraclass correlation coefficient (ICC). We aim to recruit a total sample size of 76 individuals for the trial.

#### Recruitment

Potentially eligible participants will be recruited by approaching consultants. They will be provided with a summary information sheet (see online supplementary appendix 1) and asked to reply with a list of patients under their care, who may be eligible to take part in the study and who have capacity to consent. Potentially eligible patients will be approached initially via their care team and invited to attend a primary meeting with the research team. After explaining the purpose of the meeting, each patient will be provided with a copy of the participant information sheet (PIS) and consent form (see online supplementary appendices 2 and 3). The PIS will detail the aim of the study and what their participation will involve and will inform them about how their data are kept confidential, their rights to withdraw and what will be carried out with the results. A minimum of 24 hours will be allowed for each patient to read through the information sheet and sign the consent form if they wish. Patient who are prepared to take part will be invited to attend a second meeting where the PIS will be explained to them thoroughly, any questions are answered and the written consent form is signed if it has not been already.

10.1136/bmjopen-2016-012710.supp1 Supplementary appendices

#### Withdrawal of participants

Participants may be withdrawn from the study either at their own request or at the discretion of the investigator. If a patient is withdrawn from the study, consent will be sought from them to continue to obtain outcome data via their care team. Participants will be made aware that withdrawal will not affect their future care and that they should withdraw any previously collected data will not be erased and may be used in the final analysis.

#### Blinding and allocation concealment

This is an open label study.

#### Objectives and outcomes

The primary outcome is the proportion of people in open employment at 12-month follow-up. Open employment is defined as having a job for at least 1 day, paying at least the minimum wage in a mainstream setting and not specifically for people with disability or special needs. In order to capture other vocational aspects of employment, we will consider the following secondary outcomes: number of hours worked, number of days employed, number of hours spent in educational activities and number of days in education. We will also measure individual's self-worth, social integration, mental health, quality of life and recidivism.

Assessment of participants will take place in community forensic mental health team sites or their home address at baseline, 6 months and 12 months. Data collection will be completed by a research assistant using the following measures.

#### Baseline

1. Information concerning sociodemographics, diagnosis and offending history will be collected using a data collection tool specifically designed for the purpose of this study. Sociodemographic data will be obtained from case files, and later verified with patients, including age, gender, work history, number of years in education, qualifications, living situation (alone, with friends/relatives, supported accommodation, inpatient, other), age at first psychiatric contact and number of admissions to mental health institutions in life time. Information on diagnosis will be determined by approaching their care team. Offending history will be determined from case files. These baseline data will be used to describe the sample and to stratify the sample by work experience prior to conducting qualitative interviews.
2. Brief Psychiatric Rating Scale (BPRS):^24^ This is a clinician/researcher rated scale which is widely used to measure psychiatric symptoms such as somatisation, anxiety, depression, hallucinations and others. It has 18 items and each item is measured on a scale of 1–7 (1=not present, 2=very mild, 3=mild, 4=moderate, 5=moderately severe, 6=severe and 7=extremely severe).
3. Social Functioning Questionnaire (SFQ):^25^ This is a clinician/researcher rated scale used to assess an individual's social functioning. It is divided into five sections (self-care skills, domestic skills, community skills, social skills and responsibility), each containing eight items rated on a scale of 1–4. Of these, 10 items are marked as ‘Index Items' which can be used to derive a global measure of social functioning.
4. Rosenberg's Self-Esteem Scale:^26^ This is a self-rated scale which measures self-esteem on 10 items. Each item is measured on a 4-point Likert scale—from strongly agree to strongly disagree.
5. Work Limitations Questionnaire (WLQ):^27^ This is a self-rated questionnaire that measures the degree with which health problems impact on specific aspects of job performance and the productivity impact of these limitations. Respondents are asked to rate their performance on 25 specific job demands, yielding 4 work limitation demands: time management, physical demand, mental/interpersonal demands and output demands.
6. Health-related quality of life, using SF-12v2^28^ and EQ5-D-3L:^29^ SF-12v2 is a 12-item self-rated questionnaire survey that measures functional health and well-being from the patient's perspective. EQ5-D is a self-rated measure of health status that provides a simple, generic measure of health for clinical and economic appraisal. It provides a descriptive profile and single index value for health status that can be used in economic evaluations of healthcare and to generate quality adjusted life years (QALYs) for comparative purposes.
7. Client Service Receipt Inventory (CSRI):^30^ This scale is used to capture data on recent use of health and social care services, accommodation and living situation, income, employment and benefits. Data collected using the CSRI can be used to calculate total costs of care.

#### Six-month and 12-month follow-up

1. Information about employment activities will be collected by asking participants structured questions about these activities. Data on educational activities and other vocational activities such as training and volunteering will also be collected.
2. Repeat measures for all other outcome measures, including BPRS, SFQ, Rosenberg's Self-Esteem Scale, WLQ, SF-12v2, EQ5-D and CSRI, will be obtained.
3. Reoffending data: These will be based on self-report and Police National Computer records obtained via the locally agreed protocol between the police and NHS Trust.

#### Planned analysis and study data

Analysis will be conducted on a treatment allocated basis. We will describe recruitment and retention rates, ICCs of outcome measures (primary and secondary) and patterns of missing data. We will provide means and SDs for continuous outcomes and percentage proportions for categorical outcomes with CIs. Comparisons between the two arms will be made for the outcome measures administered to estimate potential magnitude of effect.

It must be acknowledged that estimating ICCs with small numbers of participants can potentially result in biased or imprecise estimates. Because of this, the feasibility study will not be the only source to estimate ICCs. Published or unpublished data of similar outcomes in adult mental health settings in the UK will also be used to estimate ICCs.

Monitoring of study data shall include confirmation of informed consent, source data verification, data storage and data transfer procedures, local quality control check and procedures, back-up and disaster recovery of any local databases and validation of data manipulation. All data held electronically concerning the primary outcome variables will be checked against the paper record for accuracy. Ten per cent of the remaining data will be checked in the same way. Where corrections are required, these will carry a full audit trail and justification.

### Process evaluation (30 months)

Process evaluations will be carried out in parallel to the other two strands. The following three methods will be used:

1. Qualitative interviews with staff: a purposive sample reflective of a mix of backgrounds and experience will be selected for interview. Semistructured interviews will ask staff about their general views of IPS, barriers and facilitators to implementation. Interviews will continue until saturation is achieved. All interviews will be recorded and transcribed.
2. Fidelity reviews: Fidelity of the IPS model will be assessed by an external facilitator (IPS Expert) at 6, 12 and 24 months using the supported employment fidelity scale.^31^ The aim of these reviews is to assess the extent to which the services follow the eight principles of IPS and to assess how well the employment specialist functions within the service.
3. Qualitative interviews with patients: At 12 months, semistructured interviews will be completed with patients assigned to the intervention group who received IPS. A purposive sample reflecting a mix of work experience will be recruited. Specific questions will be asked about their general views of IPS, benefits and disadvantages of participating, barriers to implementation and acceptability. Interviews will continue until saturation is achieved. All interviews will be recorded and transcribed.

#### Planned analysis

Interviews will be recorded and transcribed verbatim with the interviewee's consent. The content of each transcript will be analysed using framework analyses, a validated analysis approach for policy research.^32^ The framework will be initially drafted using the key factors required to inform the ensuing study, such as barriers and facilitators. The framework will be revised iteratively as new themes appear, or original ones are removed due to lack of support by the qualitative data.

## Study management

### Research management group

A research management group has been established to manage the overall governance of the programme and day-to-day operations.

### Patient and public involvement

A patient and public involvement (PPI) reference group comprising patients, carers and representatives from Voluntary Sectors will be formed. The PPI reference group will meet quarterly throughout the duration of the programme. Group members will contribute meaningfully to all stages of the project and will provide valuable insight and advice. The PPI group will also be involved in helping to develop a leaflet and an implementation guide about IPS. Patients and carers of the group will be paid according to the Nottinghamshire Healthcare NHS Foundation Trust payment policy. Two members of the PPI will be part of the research management group and one PPI member will be involved in the process evaluation aspect of the study by conducting qualitative interviews with staff along with the research assistant, after they have been adequately trained.

### Ethics and dissemination

Should a protocol amendment be made that requires Research Ethics Committee (REC) approval, the changes in the protocol will not be instituted until the amendment and revised Informed Consent Forms (ICFs) and PIS have been reviewed and received approval from the REC and R&D departments. Should amendments be made to the final protocol which might affect a participant's participation in the study, continuing consent will be obtained using an amended ICF which will be signed by the participant. A guide to implementation of IPS in community forensic mental health settings will be developed based on the findings of this study. Final and interim reports will be prepared for project funders, the study sponsor and the clinical research network. Papers for peer-reviewed journals will be submitted for publication. Conference and event presentations will also be given.

### Adverse events

Decisions to stop the study will not be influenced by the analyses as these will be carried out at the end of the study period. However, any untoward serious incidents, including suicide and serious harm to others, will be reviewed and if there is any indication that these are linked to the intervention, the research will be stopped on the advice of the research management group.

### Records and source documents

Each participant will be assigned a trial identity code number for use on trial documents and electronic database. The list that links the identity codes to participants will be held securely by the chief investigator in accordance with the Nottinghamshire Healthcare NHS Foundation Trust Policies and procedures. The questionnaires will be treated as confidential documents and access shall be restricted to approved personnel. Electronic data will be encrypted and paper copies retained in a locked cabinet.

Source documents, which include questionnaires and interview transcriptions, shall be filed at the chief investigator’s site in a locked room. Only study staff will have access to study documentation.

### Study audit

Study conduct will be subject to systems audit of the Study Master File for inclusion of essential documents. The chief investigator, or where required, a nominated designee of the sponsor, shall carry out a site systems audit at least yearly and an audit report shall be made to the research management group.

## Discussion

This study aims to assess the feasibility of conducting a fully powered RCT to evaluate the effectiveness of IPS in improving employment rates and associated psychosocial outcomes in forensic psychiatric populations and will explore the barriers and facilitators to implementation of IPS. Findings will tell us how best to introduce IPS to forensic mental health settings and the parameters required to design a full RCT.

Based on the recruitment rate in another IPS trial in generic community mental health settings in Nottingham^22^ and feasibility criteria set out by another trial in the same service in which the feasibility study will be conducted,^33^ we proposed that a definitive trial would be considered feasible if:

1. The recruitment rate to the project is at least 50% of all referrals.
2. Fifty per cent completion rate for those assigned to the intervention is achieved.
3. Eighty per cent of those assigned to IPS will find the intervention acceptable (a score of >3 on a 5-point Likert scale indicates acceptability).
4. Fifty per cent of participants have completed all outcome measures at baseline and follow-up.

This project is designed to have an impact in the longer term, building capacity in the local community forensic service to support patients into employment beyond the life of the project. An IPS service that meets the criteria for fidelity will affect the clinical services and their personnel, as well as on the employers, with whom the service interacts. IPS fosters a culture of employability, and this is a virtuous circle: care coordinators, their managers, potential employers and commissioners grow in the conviction that anyone who wishes to work can, with suitable support, attain their ambition. Our study will provide commissioners with a full understanding of how IPS could be implemented in forensic mental health settings throughout the UK and will provide an insight into the structural-level, organisational-level, legal-level and individual-level barriers to the implementation of IPS in forensic mental health settings, in accordance with the policy climate. The proposed study is highly original and has the potential to improve the employment prospects of patients in community forensic mental health settings. By helping patients with offending histories to obtain competitive employment, they will hopefully enjoy many of the psychological and social benefits that working status entails, offering the possibility of cost savings, as people in employment reduce their use of the NHS and make progress towards greater economic independence and reduced reoffending.

## References

[1] Chanfreau J, Lloyd C, Byron C Predicting wellbeing. London: National Centre for Social Research, 2013:1–150.

[2] Waddell G, Burton K Is working good for your health and well-being? London: Department for Work and Pensions, 2006.

[3] Gulliford J, Shannon D, Taskila T Sick of being unemployed: the health issues of out of work men and how support services are failing to address them. Lancaster University: The Work Foundation, 2014.

[4] Boyce M, Secker J, Johnson R Mental health service users’ experiences of returning to paid employment. Disabil Soc 2008;23:77–88. 10.1080/09687590701725757

[5] Kinoshita Y, Furukawa TA, Omori IM Supported employment for adults with severe mental illness. Cochrane Database Syst Rev 2013;CD008297 10.1002/14651858.CD008297.pub2 24030739 PMC7433300

[6] TUC. Disability and employment: a social model study of the employment experiences of disabled people in Great Britain, with a focus on mental illness 2015 https://www.tuc.org.uk/sites/default/files/DisabilityandEmploymentReport.pdf (accessed Mar 2016).

[7] Department for Work and Pensions. The disability and health employment strategy: the discussion so far. London: Department for Work & Pensions, 2013 https://www.gov.uk/government/uploads/system/uploads/attachment_data/file/266373/disability-and-health-employment-strategy.pdf (accessed Feb 2016).

[8] Ministry of Justice and Department for Work and Pensions. Offending, employment and benefits—emerging findings from the data linkage project, Ministry of Justice ad hoc statistical bulletin. London: Ministry of Justice, 2011.

[9] Ministry of Justice. Analysis of the impact of employment on re-offending following release from custody using propensity score matching 2013 https://www.justice.gov.uk/downloads/statistics/ad-hoc/impact-employment-reoffending.pdf (accessed Mar 2016).

[10] Brooker C, Druggan S, Fox C Short-changed: spending on prison mental health care. London: Sainsbury Centre for Mental Health, 2008.

[11] Sneed Z, Koch DS, Estes H Employment and psychosocial outcomes for offenders with mental illness. Int J Psychosoc Rehabil 2006;10:103–12.

[12] Social Exclusion Unit. Reducing reoffending by ex-prisoners. London: Social Exclusion Unit, 2002.

[13] Crowther R, Marshall M, Bond G Vocational rehabilitation for people with severe mental illness. Cochrane Database Syst Rev 2001;CD003080 10.1002/14651858.CD003080 11406069 PMC4170889

[14] Burns T, Catty J, Becker T The effectiveness of supported employment for people with severe mental illness: a randomised controlled trial. Lancet 2007;370:1146–52. 10.1016/S0140-6736(07)61516-5 17905167

[15] Sainsbury Centre for Mental Health. Sainsbury centre briefing 37: doing what works: individual placement and support into employment. London: Sainsbury Centre for Mental Health, 2009.

[16] Rinaldi M, Perkins R, Glynn E Individual placement and support: from research to practice. Adv Psychiatr Treat 2008;14:50–60. 10.1192/apt.bp.107.003509

[17] Bond GR, Drake RE, Becker DR An update on randomized controlled trials of evidence-based supported employment. Psychiatr Rehabil J 2008;31:280–9. 10.2975/31.4.2008.280.290 18407876

[18] Talbot EC, Völlm B, Khalifa N Effectiveness of work skills programmes for offenders with mental disorders: a systematic review. Crimin Behav Ment Health 2015 10.1002/cbm.1981 26381597

[19] Sainsbury Centre for Mental Health. Sainsbury centre briefing 42: beyond the gate: securing employment for offenders with mental health problems. London: Sainsbury Centre for Mental Health, 2010.

[20] McMurran M, Khalifa N, Gibbon S Forensic mental health. Cullompton (Devon): Willan, 2009.

[21] Craig P, Dieppe P, Macintyre S Developing and evaluating complex interventions: new Medical Research Council guidance. BMJ 2008;337:a1655 10.1136/bmj.a1655 18824488 PMC2769032

[22] Akhtar A, Schneider J Implementing individual placement and support: the Nottingham experience. Psychiatr Rehabil J 2012;35:325–32. 10.2975/35.4.2012.325.332 22491372

[23] Eldridge S, Kerry SA Practical guide to cluster randomized trials in health services research. John Wiley & Sons, 2012.

[24] Overall JE, Gorham DR The Brief Psychiatric Rating Scale (BPRS): recent developments in ascertainment and scaling. Psychopharmacol Bull 1998;24:97–9.

[25] Tyrer P, Nur U, Crawford M Social Functioning Questionnaire: a rapid and robust measure of perceived functioning. Int J Soc Psychiatry 2005;51:265–75. 10.1177/0020764005057391 16252794

[26] Rosenberg M Society and the adolescent self image. Princeton University Press, 1965.

[27] Lerner DJ, Amick BC III, Rogers WH The Work Limitations Questionnaire: a self-administered instrument for assessing on-the-job work disability. Med Care 2001;39:72–85. 10.1097/00005650-200101000-00009 11176545

[28] Ware JE, Kosinski M, Turner-Bowker DM How to score version 2 of the SF-12v2® health survey: with a supplement documenting version 1. Lincoln (RI): QualityMetric Incorporated, 2002.

[29] EuroQol Group. EuroQol: a new facility for the measurement of health related quality of life. Health Policy 1990;16:199–208. 10.1016/0168-8510(90)90421-9 10109801

[30] Beecham J, Knapp M Costing psychiatric interventions. In: Thornicroft G, ed.. Measuring mental health needs. 2nd edn London: Gaskell, 2001:200–24.

[31] Bond GR, Peterson AE, Becker DR Validation of the revised individual placement and support fidelity scale (IPS-25). Psychiatr Serv 2012;63:758–63. 10.1176/appi.ps.201100476 22660842

[32] Ritchie J, Spencer L Qualitative data analysis for applied policy research. In: Bryman A, Burgess RG, eds. Analyzing qualitative data. London: Sage, 1994:173–19.

[33] McMurran M, Cox WM, Whitham D The addition of a goal-based motivational interview to treatment as usual to enhance engagement and reduce dropouts in a personality disorder treatment service: results of a feasibility study for a randomized controlled trial. Trials. 2013;14:1. 23414174 10.1186/1745-6215-14-50 PMC3598789

