# Supplementary material for: Individual placement and support (IPS) for patients with offending histories: the IPSOH feasibility cluster randomised trial protocol
Source: BMJ Open. 2016 Jul 22;6(7):e012710. doi: 10.1136/bmjopen-2016-012710 (PMC4964185; doi:10.1136/bmjopen-2016-012710)
Supplement: Supplementary appendices [file bmjopen-2016-012710supp_appendices.pdf]

Appendices

Appendix 1

Summary Information Sheet for Consultant Psychiatrists

Study title

Individual Placement and Support for Patients with Offending Histories.

Name of Researchers

Dr Najat Khalifa, Professor Birgit Völlm, Professor Justine Schneider, Mr Peter Bates, Ms Yvonne Bird, Dr Dawn-Marie Walker, Mr David Davies, Dr Julie Hall and Miss Emily Talbot.

We are conducting a study examining the feasibility of implementing Individual Placement and Support (IPS) in community forensic mental health settings. We are asking for your consent to approach patients under your care. Below details what the study will involve and who is organising this. Please take your time to read through this information and ask if anything is unclear.

What is the purpose of the study?

The overall aim of the study is to assess the feasibility of conducting a Cluster Randomised Controlled Trial (RCT) to evaluate the effectiveness of Individual Placement and Support (IPS) in improving employment rates and associated psychosocial outcomes in forensic psychiatric populations. IPS, a form of supported employment, aims to get people who want to work into competitive employment quickly and in accordance with their preferences. It provides individualised support, for the person and their employer, via employment specialists collocated within clinical teams. Benefits counselling is included. Besides using a range of outcome measure, we will conduct qualitative interviews with patients assigned to IPS to examine their views about IPS, exploring structural, legal and organisational barriers and facilitators to implementation.

Who is organising and funding the research?

This research is being carried out as collaboration between the Nottinghamshire Healthcare NHS Foundation Trust and the Division of Psychiatry and Applied Psychology, University of Nottingham. This study is funded by National Institute for Health Research via the Research for Patient Benefit Programme. More information about this programme can be found at: http://www.nihr.ac.uk/funding/research-for-patient-benefit.htm.

What would be involved?

This is a feasibility cluster RCT. Four clusters have been defined according to clinical services in the community forensic services of Nottinghamshire Healthcare NHS Foundation Trust. Two clusters have been randomly assigned to the intervention (IPS + treatment as usual) and two to the control group (treatment as usual). We will conduct qualitative interviews with patients assigned to IPS to examine their views about IPS, exploring structural, legal and organisational barriers and facilitators to implementation.

What are the Inclusion and Exclusion Criteria?

Patients (females and males) aged 18 and over who are currently on the caseload of the community forensic services, including personality disorder services and who have an offending history will be eligible to take part in the study.

Patients who are unable to provide informed consent, are not eligible to work in the UK, are currently in open employment or do not wish to work will be excluded from the study.

What exactly will happen during the study?

Patients assigned to the intervention will receive routine care (treatment as usual) and employment support from an employment specialist who will assess their job preferences, explore employment opportunities, and support them in applying for full or part-time jobs as part of their Care Plan. Patients assigned to the control group will receive routine care only.
The employment specialist is part of the patient's care team, and for patients assigned to IPS they will share information about their progress at work with other members of the care team. However, at no point will prospective employers be given information or told about their mental health condition and/or offending history without their permission. Disclosure of information about offending history is the responsibility of the patient and the Employment Specialist will support them in doing so and only disclose to potential employers about their mental health only if the wishes for them to be told.

For all patients, we will access their healthcare records, with their permission, to obtain specific information including age, age at first psychiatric contact, number of admissions to mental health institutions in life time, number of years in education, qualifications, living situation and work history. We will also contact the patient's consultant psychiatrist or care coordinator to obtain information about diagnosis. We will also collect data on a range of outcome measures including employment and educational activities, mental health, self-esteem, social functioning, economical outcomes and reoffending. It is also possible that certain patients assigned to IPS will be invited to take part in a qualitative study to examine their general views of IPS, exploring barriers and facilitators to implementation. Interviews will be audio recorded and each interview will last for an hour.

Confidentiality

The patients will be informed what they disclose in the study will not be discussed with anybody else unless they disclose certain information about risk of harm to themselves or others or about an unreported crime.

Withdrawal

Patients will be told they are free to withdraw at any time and without giving a reason. A decision to withdraw at any time, or not to take part, will not adversely affect them in any way and that if they withdraw, the information that has already been collected cannot be erased and may still be used in the final analysis. Patients in the intervention group will be informed that if they withdraw from the research they will not be able to continue accessing the IPS service and will instead be given the option of being referred to a contact at the Department for Work and Pensions.

Pros and Cons for patient involvement

We cannot promise the study will help individual patients but the information we get from this study will help improve the treatment of people with mental health problems who have a history of offending. Participating in this research should not cause patients any inconvenience, discomfort or distress.

Expenses and payments

Participants will not be paid to participate in the trial. Travel expenses will be offered for visits in excess of usual care.

What will happen to the results of the research study?

At the end of this study, a summary of our findings will be posted on the websites of Nottinghamshire Healthcare NHS Foundation Trust (www.nottinghamshirehealthcare.nhs.uk/) and the Institute of Mental

Health (www.institutemh.org.uk). We will also write papers for conferences and journal publications. We can also provide verbal and written feedback for individuals.

Who has reviewed the study?

This study has been reviewed and given favourable opinion by Nottingham Research Ethics Committee. It has also been approved by the Research and Development Department of Nottinghamshire Healthcare NHS Foundation Trust.

Chief Investigator: Dr Najat Khalifa**,** Institute of Mental Health**,** University of Nottingham Innovations Park**,** Triumph Road, Nottingham, NG7 2TU. Tel: 01158231269, Email: najat.khalifa@nottingham.ac.uk.

Research Assistant: Miss Emily Talbot, Institute of Mental Health, University of Nottingham Innovations Park, Triumph Road, Nottingham, NG7 2TU. Tel: 07785525420. Email: emily.talbot@nottshc.nhs.uk

CONSENT FORM

We require consent to approach patients under your care that meet the inclusion and exclusion criteria as detailed above.

1. I consent to the research team approaching patients under my care that meet the above criteria ☐

2. I confirm the following patients meet the inclusion/exclusion criteria: ☐

______________________________	______________________________
______________________________	______________________________
______________________________	______________________________
______________________________	______________________________
______________________________	______________________________
______________________________	______________________________
______________________________	______________________________
______________________________	______________________________
______________________________	______________________________
______________________________	______________________________

--------------------------------------	--------------------------	---------------------------
Consultant Psychiatrist	Date	Signature
--------------------------------------	------------------------	---------------------------
Researcher	Date	Signature

Appendix 2

Participant Information Sheet (IPS)

Study title

Individual Placement and Support for Patients with Offending Histories

Name of Researchers

Dr Najat Khalifa
Professor Birgit Völlm
Professor Justine Schneider
Mr Peter Bates
Ms Yvonne Bird
Dr Dawn-Marie Walker
Mr David Davies
Dr Julie Hall
Miss Emily Talbot

We would like to invite you to take part in a research study whose purpose is to increase access to employment. Before you decide, you need to understand why the research is being done and what it would involve for you. Please take time to read this information sheet carefully before deciding whether to participate. Talk to others about the study if you wish and/or ask a member of staff or the Researcher for more information.

Why have I been invited?

You have been invited to take part because you have been identified by your consultant psychiatrist or care coordinator as someone who is not currently in paid employment. If you agree to take part we will ask you to sign a consent form by meeting with the Research Assistant (Miss Emily Talbot) who will also be able to answer any questions that you may have about the study.

What is the purpose of the study?

The purpose of this study to assess whether Individual Placement and Support (IPS), a form of supported employment, can be introduced in forensic mental health settings. This study will enable more people who use community mental health services to gain paid work by offering them support through an Employment Specialist. Employment may not be among your short term goals right now, but most people hope that they will have a job of their choice one day.

Do I have to take part?

Whether or not you take part is for you to decide but we can only offer the IPS service to you if you agree to participate in the research study. If you agree to take part, we will ask you to sign a consent form. If you change your mind, you are free to withdraw at any point, without giving a reason. This would not affect the standard of care you receive but once you withdraw you will no longer be able to access the IPS service. Instead you will have the option of being referred to a contact at the Department for Work and Pensions. If you withdraw, the information that has already been collected cannot be erased and may still be used in the final analysis.

What will I have to do if I participate?

With your permission, we will access your healthcare records to obtain information about your education, living situation, offending history and work history. We will also contact your

consultant psychiatrist or care coordinator to obtain information about your mental health condition. You will receive employment support from an Employment Specialist who will assess your job preferences, explore employment opportunities and support you in applying for full or part-time jobs as part of your Care Plan.

You will also be asked to meet with the researcher (Miss Emily Talbot) three times for an interview where the researcher will complete paper-based questionnaires; this will be at the start of the research, after six months and then again at twelve months. This interview will not be audio recorded and will last approximately one hour. You can choose to have this interview take place at the community mental health site that you visit or at your home address, depending on what is convenient for you. The researcher will ask you questions about how you are feeling, including information about your health and how you are getting on with finding or continuing your employment. It is also possible that you may be invited to take part in an in-depth interview which will be audio recorded. During this interview the researcher (Miss Emily Talbot) and another member of the research team will ask you about your experience and satisfaction with the process of employment support that you have received, and how it can be improved. Again, this interview will take place in a venue of your choice. That is a maximum total of four contacts over one year.

What exactly will happen during the study?

You will receive support from the Employment Specialist. This will involve you having regular meetings with them at a time and venue convenient to you. They will support you with your job search, finding out what kind of work is available and matching this to your preferences and skills. If you wish them to meet with your employer or to negotiate any aspect of the work on your behalf, the employment specialists may do this, but only if you agree. Once you are in employment, if you face any problems, they will help you deal with them. All these meeting with the employment specialist will last about 45 minutes. The treatment you receive from the community forensic team will not be affected in any way.

The Employment Specialist is part of your care team and they will share information about your progress at work with members of the care team. However, at no point will prospective employers be given information or told about your mental health condition and/or offending history without your permission. It is your responsibility to tell prospective employers about your offending history. The Employment Specialist will support you in doing so and only disclose information about your mental health to potential employers if you wish them to be told.

What are the possible benefits of taking part?

We cannot promise that the study will help you but receiving employment support may help fulfil your employment aspiration as a part of your recovery. Additionally, the information we get from this study will help improve the treatment of people with mental health problems.

What are the possible disadvantages of taking part?

Participating in this research should not cause you any inconvenience, discomfort or distress. Some of the questions may be quite personal but any information you share will be kept strictly confidential and anonymous.

Expenses and payments

You will not be paid to participate in the study. Travel expenses will be offered for visits in excess of usual care.

What happens when the research study stops?

You will receive ongoing support from your care team even after the study stops.

What if relevant new information becomes available?

The Chief Investigator will inform you of any relevant information that becomes available during the course of the study and will discuss with you whether you wish to continue with the study.

What if there is a problem?

If you have any questions, concerns, or complaints about this study, you can contact any of the people listed at the bottom of this page or the Patient Advice and Liaison Service (PALS). You can contact PALS by phoning them on 0800 **015 3367.**

Will my taking part in the study be kept confidential?

Yes. Any information you share will be kept strictly confidential and anonymous. This means that the interview data will only be accessible to the research team and you will not be recognisable in research reports or publications. We cannot promise to keep confidential any information relating to criminal activities nor to immediate risk of harm to you or to other people. In such cases the researcher would inform your care coordinator or another relevant individual in authority.

What will happen to the results of the research study?

At the end of this study, a summary of our findings will be posted on the websites of Nottinghamshire Healthcare NHS Foundation Trust (www.nottinghamshirehealthcare.nhs.uk/) and the Institute of Mental Health (www.institutemh.org.uk). We will also write papers for conferences and journal publications. We can also provide verbal and written feedback for individuals.

Who is organising and funding the research?

This research is being carried out as collaboration between the Nottinghamshire Healthcare NHS Foundation Trust and the Division of Psychiatry and Applied Psychology, University of Nottingham. This study is funded by National Institute for Health Research via the Research for Patient Benefit Programme.

Who has reviewed the study?

All research in the NHS is looked at by independent group of people, called a Research Ethics Committee to protect your safety, rights, wellbeing and dignity. This study has been reviewed and given favourable opinion by Nottingham Research Ethics Committee 1.

Further information and contact details

If you would like more information about this research project or have any questions or concerns, please contact the Researcher or Chief Investigator. Their contact details are provided below.

Miss Emily Talbot, Institute of Mental Health, University of Nottingham Innovations Park, Triumph Road, Nottingham, NG7 2TU**. Tel: 07785525420**. Email: **emily.talbot**@nottshc.nhs.uk

Dr Najat Khalifa (Chief Investigator). Institute of Mental Health, University of Nottingham Innovations Park, Triumph Road, Nottingham, NG7 2TU. Tel: 0115 8231269.
Email: najat.khalifa@nottshc.nhs.uk

Thank you for taking the time to read this information

CONSENT FORM

Title of Study:
Individual Placement and Support for people with Offending Histories

REC ref: (15/EM/0253)

Chief Investigator: Dr Najat Khalifa

Please initial box

Name of Participant:

1. I confirm that I have read and understood the information sheet version number 4 dated 25/02/2016 for the above study and have had the opportunity to ask questions. ☐

2. I understand that my participation is voluntary and that I am free to withdraw at any time, without giving any reason, and without my medical care or legal rights being affected. I understand that should I withdraw, I will no longer have access to IPS services and that the information collected so far cannot be erased and that this information may still be used in the project analysis. ☐

3. I understand that all data collected in the study may be looked at by authorised individuals from the University of Nottingham, Research and Development Department of Nottinghamshire Healthcare NHS Foundation Trust and the research team. I give permission for these individuals to collect, store, analyse and publish information obtained from my participation in this study. I understand that my personal details will be kept confidential. ☐

4. I understand that my healthcare records will be looked at by the Chief Investigator or a researcher working under his supervision. I give permission for these individuals to collect specific data from these records about me including age, age at first psychiatric contact, number of admissions to mental health institutions in life time, number of years in education, qualifications, living situation, offending history and work history. ☐

5. I understand that my consultant psychiatrist will be contacted by the Chief Investigator or a researcher working under his supervision to obtain specific information about my mental health condition. I give permission for the consultant psychiatrist to disclose information to these individuals about my mental health condition. ☐

6. I understand that any information I share will be kept strictly confidential and anonymous unless I disclose certain information about myself or someone else being harmed or about an unreported crime. In such cases the researcher will inform my care coordinator or another relevant individual in authority. ☐

7. I agree to take part in the above study. ☐

------------------------------------	-------------------------	------------------------------
Name	Date	Signature
------------------------------------	------------------------	-------------------------------
Name of Person taking consent<br>(if different from researcher)	Date	Signature
-----------------------------------	------------------------	---------------------------------
Researcher	Date	Signature

3 copies: 1 for participant, 1 for the project notes and 1 for patient’s healthcare records.

Appendix 3

Participant Information Sheet (Controls):

Study title

Individual Placement and Support for Patients with Offending Histories

Name of Researchers
Dr Najat Khalifa
Professor Birgit Völlm
Professor Justine Schneider
Mr Peter Bates
Ms Yvonne Bird
Dr Dawn-Marie Walker
Mr David Davies
Dr Julie Hall
Miss Emily Talbot

We would like to invite you to take part in a research study whose purpose is to increase access to employment. Before you decide, you need to understand why the research is being done and what it would involve for you. Please take time to read this information sheet carefully before deciding whether to participate. Talk to others about the study if you wish and/or ask a member of staff or the Researcher for more information.

Why have I been invited?

You have been invited to take part because you have been identified by your consultant psychiatrist or care coordinator as someone who is not currently in paid employment. If you agree to take part we will ask you to sign a consent form by meeting with the Research Assistant (Miss Emily Talbot) who will also be able to answer any questions that you may have about the study.

What is the purpose of the study?

The purpose of this study to assess whether Individual Placement and Support (IPS), a form of supported employment, can be introduced in forensic mental health settings. Whilst IPS is not being tested at your location, it is hoped that by comparing your progress against those in other locations with IPS will allow better provision of employment support during treatment in the future. Employment may not be among your short term goals right now, but most people hope that they will have a job of their choice one day.

Do I have to take part?

Whether or not you take part is for you to decide. If you agree to take part, we will ask you to sign a consent form. If you change your mind, you are free to withdraw at any point, without giving a reason. This would not affect the standard of care you receive. If you withdraw, the information that has already been collected cannot be erased and may still be used in the final analysis.

What will I have to do if I participate?

With your permission, we will access your healthcare records to obtain information about your education, living situation, offending history and work history. We will also contact your consultant psychiatrist or care coordinator to obtain information about your mental health condition. You will also be asked to meet with the researcher (Miss Emily Talbot) three times for an interview where the researcher will complete paper-based questionnaires; this will be at the start of the research, after six months and then again at twelve months. This interview will not be audio recorded and will last approximately one hour. You can choose to have this interview take place at the community mental health site that you visit or at your home address, depending on what is convenient for you. The researcher will ask you questions about how you are

feeling, including information about your health and how you are getting on with finding or continuing your employment.

What exactly will happen during the study?

You will be part of the control group which means that the treatment you receive from the community forensic team will not be affected in anyway. You will only be meeting with the Research Assistant three times for an interview as detailed above.

What are the possible benefits of taking part?

We cannot promise the study will help you but the information we get from this study will help improve the treatment of people with mental health problems who have an offending history.

What are the possible disadvantages of taking part?

Participating in this research should not cause you any inconvenience, discomfort or distress. Some of the questions may be quite personal but any information you share will be kept strictly confidential and anonymous unless you disclose certain information about yourself or someone else being harmed or about an unreported crime.

Expenses and payments

You will not be paid to participate in the study. Travel expenses will be offered for visits in excess of usual care.

What happens when the research study stops?

You will receive ongoing support from your care team even after the study stops.

What if relevant new information becomes available?

The Chief Investigator will inform you of any relevant information that becomes available during the course of the study and will discuss with you whether you wish to continue with the study.

What if there is a problem?

If you have any questions, concerns, or complaints about this study, you can contact any of the people listed at the bottom of this page or the Patient Advice and Liaison Service (PALS). You can contact PALS by phoning them on 0800**0153367.**

Will my taking part in the study be kept confidential?

Yes. Any information you share will be kept strictly confidential and anonymous. This means that the interview data will only be accessible to the research team and you will not be recognisable in research reports or publications. We cannot promise to keep confidential any information relating to criminal activities nor to immediate risk of harm to you or to other people. In such cases the researcher would inform your care coordinator or another relevant individual in authority.

What will happen to the results of the research study?

At the end of this study, a summary of our findings will be posted on the websites of Nottinghamshire Healthcare NHS Foundation Trust (www.nottinghamshirehealthcare.nhs.uk/) and the Institute of Mental

Health (www.institutemh.org.uk). We will also write papers for conferences and journal publications. We can also provide verbal and written feedback for individuals.

Who is organising and funding the research?

This research is being carried out as collaboration between the Nottinghamshire Healthcare NHS Foundation Trust and the Division of Psychiatry and Applied Psychology, University of Nottingham. This study is funded by National Institute for Health Research via the Research for Patient Benefit Programme. More information about this programme can be found at: http://www.nihr.ac.uk/funding/research-for-patient-benefit.htm.

Who has reviewed the study?

All research in the NHS is looked at by independent group of people, called a Research Ethics Committee to protect your safety, rights, wellbeing and dignity. This study has been reviewed and given favourable opinion by Nottingham Research Ethics Committee 1.

Further information and contact details

If you decide to take part in this study you will be given a copy of this information sheet to keep and a copy of the consent form which you have signed. If you decide to take part in this study then you should contact our Researcher (name, details given below) or return the enclosed contact slip giving us your contact details so that we can contact you.

If you would like more information about this research project or have any questions or concerns, please contact the Researcher or Chief Investigator. Their contact details are provided below.

Miss Emily Talbot, Institute of Mental Health, University of Nottingham Innovations Park, Triumph Road, Nottingham, NG7 2TU. Tel: **07785525420.** Email: **emily.talbot@nottingham.ac.uk**

Dr Najat Khalifa (Chief Investigator). Institute of Mental Health, University of Nottingham Innovations Park, Triumph Road, Nottingham, NG7 2TU. Tel: 0115 8231269.
Email: najat.khalifa@nottingham.ac.uk

Thank you for taking the time to read this information

CONSENT FORM

Title of Study:

Individual Placement and Support for people with Offending Histories

REC ref: (15/EM/0253)

Chief Investigator: Dr Najat Khalifa

Name of Participant: **Please initial box**

1. I confirm that I have read and understood the information sheet version number 4dated 25/02/2016 for the above study and have had the opportunity to ask questions.

2. I understand that my participation is voluntary and that I am free to withdraw at any time, without giving any reason, and without my medical care or legal rights being affected. I understand that should I withdraw then the information collected so far cannot be erased and that this information may still be used in the project analysis.

3. I understand that all data collected in the study may be looked at by authorised individuals from the University of Nottingham, Research and Development Department of Nottinghamshire Healthcare NHS Foundation Trust and the research team. I give permission for these individuals to collect, store, analyse and publish information obtained from my participation in this study. I understand that my personal details will be kept confidential.

4. I understand that my healthcare records will be looked at by the Chief Investigator or a researcher working under his supervision. I give permission for these individuals to collect specific data from these records about me including age, age at first psychiatric contact, number of admissions to mental health institutions in life time, number of years in education, qualifications, living situation, offending history and work history.

5. I understand that my consultant psychiatrist will be contacted by the Chief Investigator or a researcher working under his supervision to obtain specific information about my mental health condition. I give permission for the consultant psychiatrist to disclose information to these individuals about my mental health condition.

6. I understand that any information I share will be kept strictly confidential and anonymous unless I disclose certain information about myself or someone else being harmed or about

an unreported crime. In such cases the researcher will inform my care coordinator or another relevant individual in authority

7. I agree to take part in the above study. ☐

--------------------------------------	--------------------------	-------------------------------
Name	Date	Signature
--------------------------------------	-------------------------	--------------------------------
Name of Person taking consent (if different from researcher)	Date	Signature
-------------------------------------	-------------------------	----------------------------------
Researcher	Date	Signature

3 copies: 1 for participant, 1 for the project notes and 1 for patient's healthcare records.
